# The Subjective Influence of the Study of Medicine

**Published:** 1918-09-07

**Authors:** 


### THE SUBJECTIVE INFLUENCE OF THE STUDY OF MEDICINE.

It is a reasonable and proper question for anyone entering on a medical career to ask, What return may I expect from my work? Not less reasonable and proper is it that such an inquiry should include an examination of the financial prospect. The struggle for existence is imposed on most of us, and even a larger success of this order implies no unworthy ambition. Medicine professes no way to a ready and great fortune, but to diligence and thoroughness it guarantees at least a modest competence; if the making of money is the principal aim, the searcher had better turn elsewhere. Another aspect of the preliminary survey here suggested is the educational value of medical study and practice. What is the mental discipline and influence of such a life on those who adopt it? Does it encourage or discourage the development of desirable personal qualities? Is it or is it not a training-school in which are provided influences that promote the full stature of manhood? What exactly is its trend and tendency on those who live in its environment? Such questions may appear to have a selfish quality, but the selfishness is an enlightened and reasonable one. The individual has a right to select, so far as the choice is open to him, the calling which gives him the most congenial atmosphere and the best opportunities for self-realisation. Even apart from his own concern in the choice, it is in the interest of the community that he should be largely moved by this consideration. That a man ought to desire to serve others does not imply that he ought not to desire to serve himself. The personal motive is not an ignoble one, and it is apt to be enduring. With it may be associated other services in due order and perspective, but let not these be exalted at the expense of the individual claim. Both the one and the other may be kept in view, and each should get its fair position in the debate. Now, in exhortations not a few, the student has been invited to consider the "nobility" of his calling and how large will be his opportunities for the exercise of his altruistic faculties. Equally he is told, and we accept the conclusion, that a money reward ought not to be his first or chief consideration. There is no need to quarrel with these hard-worked platitudes, though they are apt to lose touch with actuality. But the student, while contemplating the chance of ministering to the needs of others, may quite reasonably consider his own need, and may ask what in this respect a medical atmosphere is going to do for him. If it will not give him a fortune in the material sense, will it train his mind and manhood and give him a capacity "to see life steadily and to see it whole"? The subjective influence of medical study is thus quite an appropriate motive to present to those who are asking themselves whether medicine is a profession that can offer them a satisfying and helpful career.

What then is the play of the forces which may be said to be more or less peculiar or special to the life of the medical practitioner? The first of these may truly be said to be a wide education in the appreciation of evidence or testimony. Possibly it may be remarked that the lawyer can dispute this position with the doctor or that the scientific atmosphere generally is able to make an equal claim with that of medicine. But there are differences. The lawyer, it is true, is concerned greatly with evidence, but it is evidence according to a particular code or standard. No doubt for forensic purposes such a code or standard is advisable or necessary, but in the nature of things it is exclusive and in a degree artificial. It pushes out of sight contributions which do not satisfy its regulations, and its inquiries are therefore in a corresponding degree limited. The question to be debated is not an unrestricted problem where all the facts and considerations may be reviewed, but a discussion restricted within limits formed by the legal definition of evidence. Not what is morally or logically relevant is admitted, but only what is legally relevant. Not justice in the widest sense is the aim, but justice according to law. Further, for all practical purposes, legal issues are closed by a definite conclusion in one direction or the other, and for various reasons this is a very necessary arrangement. But "Guilty" and "Not guilty" are obviously not the only logical comments on a debated proposition. Hesitations and uncertainties and degrees of doubt are among the possibilities, though they may not be formulated in the legal finding. In short, what is evidence in the legal sense in any particular issue is determined by rules formulated in advance, and the

**Subjective Influence of Study of Medicine**—(*cont.*).

conclusion, whatever it be, has the superficially attractive characteristics of the absolute proposition.

Very different in both of these respects is the discussion of medical issues. Here are no *a priori* rules fixing according to a prepared scheme what is and what is not evidence. The field of inquiry is unrestricted and testimony from all sources is to be welcomed. The single aim is the discovery of the truth and whatever may aid this aim is of service. No predetermined exclusions and no artificial values are admitted. Each piece of testimony stands on its own merits, and its worth and significance have to be judged by this criterion alone. The consequences, so far as the practitioner is concerned, are two in number. First he lives under influences which promote the open mind and encourage freedom of inquiry, and in the second place he is stimulated to the endeavour to judge constantly the worth of testimony. The testimony may be provided by his own observations on his patients, and with this runs a sense of the necessity of making these observations complete and accurate and exact. To be reliable and of value in the investigations in which he is concerned, his records must possess a cold impartial quality absolutely uninfluenced by any desired or expected result, and if he fails in this respect experience will provide him with some sharp checks. On the other hand, some of the testimony which comes before him presents itself in the shape of the statements of others—authorities, real or professed, who write books, the experiences related by patients, and the descriptions provided by anxious relatives or friends. Here, indeed, is a full education in the capacity and value of human testimony, and the exercise of judgment in this respect is fully promoted. If the practitioner learns by sad experience that a severe accuracy is not a widespread quality, and that exaggeration and a love of the dramatic are prominent human traits, this is a useful lesson in life, and it provides an influence which should help to keep his own contributions within the limits of a disciplined veracity. He has not, like the lawyer, rules to direct him, but neither has he chains to limit his freedom, and the arrangement is not without its compensations. Yet, again, there exists for him the opportunity of the proposition which can neither be proved nor disproved. No force compels him to vote either " Aye " or " No " when the available evidence leaves his mind uncertain. On the contrary, loyalty to truth urges him to hold his opinion in reserve until further light can be obtained, and he is led to recognise that the area within which confident knowledge is possible to him is but a limited one. He finds out that what many people profess as opinions are mere tastes, or prejudices, or quotations, and have no association whatever with an investigation of evidence. These serve well the social chatter of the hour, and are achieved without intellectual enterprise or labour. But for a medical judgment personal knowledge and strict inquiry are essential, and there is no real attainment apart from these qualities. It may be quite true that a contemplation of the limited character of the field within which personal knowledge and confident opinion are possible exercises a somewhat depressing influence on the mind. But the position has the firm ground of accuracy, and, moreover, it exercises a constant stimulus to endeavour. The unknown and the uncertain are on every hand, but they invite to enterprise and effort. Not much, perhaps, can be done, but at least something may be attempted. And with the stimulus is the reflection that no exhaustion of the field is possible. The practitioner of medicine need never fear that he will be compelled to weariness because there are no more worlds to conquer. He may have at the end to confess that he has been an unprofitable servant, but his failure will not be due to lack of opportunity. He may recognise the task as beyond the limits of the days allotted to him, but he will never lack an opening for adventure.

In the respects just enumerated medicine may be said to share with other departments of science a generous though severe educational training in the appraisement of evidence, and in the practice of intellectual honesty. The problem to be resolved at the bedside of a patient, so far as it relates to an intellectual judgment, is governed by exactly the same demands as those which are in force for a physical or chemical inquiry conducted in a laboratory. The evidence in each instance has to be collected and weighed in accordance with the strict rules of science. But in medicine there is something more. The scientific problem is there, but it is associated with a question of immediate human interest. There exists, indeed, the cold, hard, diagnostic issue, and no smooth and comfortable words can conjure this away; it demands the satisfaction of intellectual honesty. The subject of the inquiry, however, is a human being with the interests and longings that in greater or less measure form the common lot. The medical practitioner has this consideration in his life as well as the more rigidly scientific one. He is in touch constantly with the sense of tears in human affairs, and no scientific inquirer so much as he has the broad view of life pressed on his attention. If he is urged constantly to a strict accuracy in observation and conclusion, he is not the less moved to the cultivation of understanding and sympathy. It is to the heart as well as to the head that the appeal is made, and the whole man, not merely his intellectual faculties, is encouraged to a liberal growth and a generous development.

Closely connected with educational training in the value of evidence and in the nature of opinion is the statement or profession of knowledge, and here medical influences, if not without temptation to the wrong direction, afford an opportunity for the cultivation of a high standard. The practitioner knows quite well his limitations and his incapacities, and equally he knows that the public largely attribute to him powers and abilities which he does not possess and to which medicine

**Subjective Influence of Study of Medicine**—(*cont.*).

makes no claim. Under the influence of such expectations, and by the pressure of practical events, there is obviously an influence which inclines men to admit or even to advance unreal pretensions. To hint a doubt or to admit a hesitation may be construed as a sign of weakness in an oracle more or less generally credited with the gift of omniscience. There is thus popular pressure tending to a departure from strict accuracy of statement, and to yield to this is to enter on a slippery slope. This is the dangerous departure that challenges the habit of intellectual honesty, and the downward path is an easy one. But in opposition to this unhappy and fatal failure is the whole force of scientific teaching and mode of thought. As he has learned to think honestly the practitioner will learn to speak honestly and not to allow uncertainties to colour themselves with a cheap and popular disguise. Reasons for action in the practical sphere there may be, even though the doctrinal question may be yet uncertain, and the two areas must not be confounded. In the one is abundant stimulus to clear decision and prompt action on the basis of probabilities, while in the other the claim is for a resolute determination to refuse to advance beyond admissions justified by evidence and to keep an open mind to new facts and to the modifications of opinion which these demand. Much more might readily be said on what is really a very wide topic. Here the attempt is merely to advance some of the grounds on which medicine may be presented as a career which offers among its rewards opportunities for mental self-discipline and the prospect of a continuous and enduring liberal education.

